# Improved expansion of equine cord blood derived mesenchymal stromal cells by using microcarriers in stirred suspension bioreactors

**DOI:** 10.1186/s13036-019-0153-8

**Published:** 2019-03-21

**Authors:** Erin L. Roberts, Tiffany Dang, Sarah I. M. Lepage, Amir Hamed Alizadeh, Tylor Walsh, Thomas G. Koch, Michael S. Kallos

**Affiliations:** 1 Pharmaceutical Production Research Facility, Schulich School of Engineering, University of Calgary, 2500 University Dr. NW, Calgary, AB T2N 1N4 Canada; 2 Biomedical Engineering Graduate Program, University of Calgary, 2500 University Dr. NW, Calgary, AB T2N 1N4 Canada; 3 Department of Biomedical Sciences, Ontario Veterinary College, University of Guelph, Gordon St, Guelph, ON N1G 2W1 Canada; 4 Department of Chemical and Petroleum Engineering, Schulich School of Engineering, University of Calgary, 2500 University Dr. NW, Calgary, AB T2N 1N4 Canada

## Abstract

Equine mesenchymal stromal cells (MSCs) are increasingly investigated for their clinical therapeutic utility. Such cell-based treatments can require cell numbers in the millions or billions, with conventional expansion methods using static T-flasks typically inefficient in achieving these cell numbers. Equine cord blood-derived MSCs (eCB-MSCs), are promising cell candidates owing to their capacity for chondrogenic differentiation and immunomodulation. Expansion of eCB-MSCs in stirred suspension bioreactors with microcarriers as an attachment surface has the potential to generate clinically relevant numbers of cells while decreasing cost, time and labour requirements and increasing reproducibility and yield when compared to static expansion. As eCB-MSCs have not yet been expanded in stirred suspension bioreactors, a robust protocol was required to expand these cells using this method. This study outlines the development of an expansion bioprocess, detailing the inoculation phase, expansion phase, and harvesting phase, followed by phenotypic and trilineage differentiation characterization of two eCB-MSC donors. The process achieved maximum cell densities up to 75,000 cells/cm^2^ corresponding to 40 million cells in a 100 mL bioreactor, with a harvesting efficiency of up to 80%, corresponding to a yield of 32 million cells from a 100 mL bioreactor. When compared to cells grown in static T-flasks, bioreactor-expanded eCB-MSC cultures did not change in surface marker expression or trilineage differentiation capacity. This indicates that the bioreactor expansion process yields large quantities of eCB-MSCs with similar characteristics to conventionally grown eCB-MSCs.

## Introduction

With nearly one million domestic horses in Canada, the horse industry contributes $19 billion annually to the Canadian economy [1]. However, $259 million is spent annually in Canada on equine veterinary services [1], with orthopedic injuries being the leading cause of loss of performance in horses [2]. Conventional treatments for orthopedic injuries in horses have been found to be ineffective, requiring lengthy recovery times and a 40–60% risk of re-injury [3]. Mesenchymal stromal cell (MSC) injections have been found to be a promising treatment option for orthopedic injuries in horses [4, 5]. Equine umbilical cord blood-derived MSCs (eCB-MSC) are attractive clinical candidates due to their non-invasive procurement, high proliferation rates and chondrogenic potential [6]. MSC-based treatments can require up to 10^9^ cells per patient [7]. Currently, eCB-MSC are isolated and expanded in conventional culture vessels under static culture conditions. However, this method is recognized as labour intensive, expensive, has low reproducibility, and is associated with a high risk of contamination. There is currently no protocol for the large-scale expansion of equine MSCs. Expansion of eCB-MSCs in stirred suspension bioreactors using microcarriers as the attachment surface has the potential to generate a clinically relevant number of cells while limiting costs and labour requirements and increasing process reproducibility.

The type of microcarrier used is critical in a bioreactor process to ensure adequate attachment and expansion of the cells. A variety of different commercially manufactured microcarriers have been tested for the expansion of MSCs, both porous and non-porous, made from a variety of different materials, with different coatings [8–11]. Chemical composition, surface topography, porosity and surface charge of the microcarrier can all affect cell attachment and have been found to be donor and cell line specific [12]. Therefore the choice of microcarrier should be optimized for a given application [13].

A stirred suspension bioreactor process can be developed in three different stages: the inoculation phase, the expansion phase, and the harvesting phase. The inoculation phase is typically described as the first 24 h of a bioprocess, during which the objective is to achieve the greatest possible attachment efficiency of cells to microcarriers. Factors that can affect attachment of cells include the confluency of the T-flask before inoculation into the bioreactors and the cell to microcarrier ratio in the bioreactor. Studies have found that lower cell confluences typically result in lower population doubling times in the subsequent growth stage [14]. Several different cell to microcarrier (MC) ratios have also been investigated for bioreactor expansion processes. Typically, with lower initial cell to MC ratios, a higher cell-fold expansion is achieved and a lower final cell density is achieved, compared to a higher cell to MC density [15, 16]. The appropriate cell to microcarrier density depends on the surface area of the microcarrier. For example, for Cytodex 3, a 4 cell/MC density is commonly used [10, 17–19].The choice of cell to MC ratio for a given process will likely be limited by other process constraints such as the availability of cell inoculum and the target cell number, time of expansion, or cost of medium.

The expansion phase is typically considered to start after the inoculation phase and continues until the required attached cell density has been obtained. An important consideration for the expansion phase is the culture medium composition as well as the medium change regime to ensure the cells have access to adequate nutrients. We (Koch) have been using a medium, based on human cord blood MSC medium, for use with eCB-MSCs, consisting of DMEM, with 30% FBS, 1% L-glutamine, and 1% antibiotics [2]. Other researchers working with eCB-MSCs have adopted this medium, with a few investigating different growth media. It is advantageous to lower the amount of FBS in the media as FBS greatly varies between lots, and therefore greatly reduces reproducibility of growth of cells. Studies using human CB-MSCs have been able to lower the FBS concentration by adding components such as platelet rich plasma, growth factors and serum albumin [20–22]. To maximize expansion of cells, a medium replacement regime is often incorporated into a cell expansion process to replace depleted nutrients and remove the build-up of growth inhibiting products. To determine the optimal medium replacement regime for a given process, it is useful to analyze the metabolic activity of the cells through the analysis of the glucose and lactate concentration of the culture medium. The effect of the culture medium used as well as the medium replacement regime is process specific and therefore should be optimized for a given process.

The agitation rate in a stirred suspension bioreactor should be optimized for each expansion method and should be investigated for various scales of growth within the process, as the appropriate agitation rate depends on the bioreactor and impeller geometry, media viscosity and density, cell type and microcarrier type. Studies have compared the use of different agitation rates for microcarrier expansion and have found that typically lower agitation rates can cause cell-microcarrier aggregates to form [23]. High agitation rates can cause high shear in the bioreactors, which can result in cells detaching from microcarriers [24, 25]. The ideal agitation rate will depend on the bioreactor scale and geometry used for a given process.

The last stage of a bioprocess is the harvesting stage, in which the cells are removed from the microcarriers, typically enzymatically, and filtered to obtain a pure cell suspension. The most common enzyme used for removal of cells from microcarriers is porcine Trypsin, in either a concentration of 0.25% or 0.05%. For a xenogeneic-free process, Trypsin is usually replaced by TrypLE, a bacterial derived recombinant substitute for porcine Trypsin. Typically, the cells are exposed to the enzyme for 5–15 min, however studies have used exposure times up to 1 h.

We hypothesized that eCB-MSCs can be expanded on microcarrier in bioreactors. Our objectives were to determine an expansion process for this specific cell type and determine if the process altered cell phenotype and in vitro functionality compared to static expanded cells.

## Methods and materials

### Study design

**Fig. 1:**
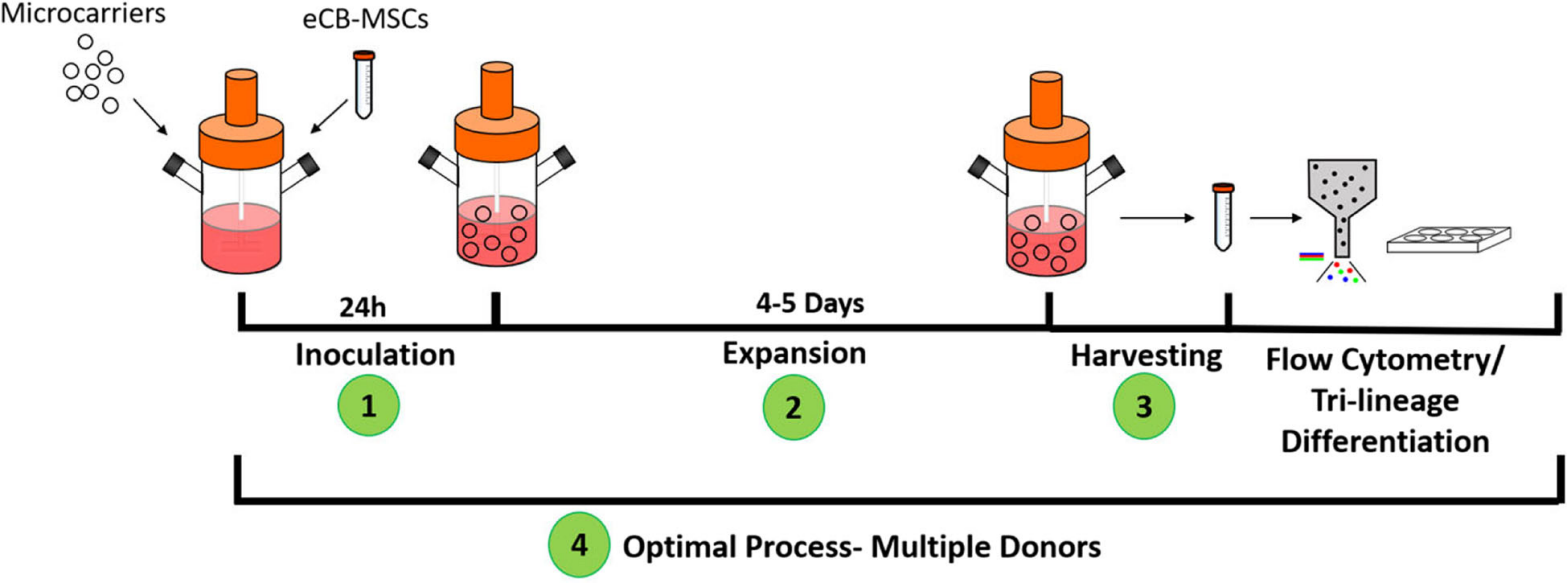
Experimental design for the expansion of eCB-MSCS on microcarriers in bioreactors. The process was developed in three phases: inoculation, expansion, and harvesting. The optimal process was then used to expand three different donor cells and characterization was performed on two donors

The experimental design for the process development is shown in Fig. 1. The process was developed in 3 different phases, 1. Inoculation phase, 2. Expansion phase, 3. Harvesting phase, followed by testing of the developed process on three different donors, including characterization of the cells after expansion.

### Cell source

Cord blood of three different foals was isolated immediately after birth and the eCB-MSCs were isolated as described previously [26]. The donors are referred to as Donor 1409, isolated from a male Quarter Horse; Donor 1201, isolated from a male Thoroughbred; and Donor 1412, isolated from a female Quarter Horse. A cell bank of eCB-MSCs was created by expanding the cells in static culture. Donor 1409 cells at passage 10 were used throughout the microcarrier screening, and inoculation, expansion, and harvesting phase process development. All three donors were then used in the final stage of the study to determine the robustness of the developed process, followed by phenotypic and tri-lineage characterization of Donors 1409 and 1201. In the final stage of the study, Donor 1409 and 1412 were at passage 10 during the first passage, and passage 11 during the second passage. Donor 1201 was at passage 8 during the first passage and passage 9 during the second passage.

### Culture media

Two different culture media were used for the expansion of the eCB-MSCs. They will be referred to as either 30%FBS-0bFGF or 10%FBS-5bFGF. The 30%FBS-0bFGF medium consisted of DMEM with 1.0 g/L glucose (Lonza Cat# 12-707F), 30% FBS (Sigma Cat #: F1051, Lot# 16c422), 2.0 mM L-Glutamine (Lonza Cat #:17-605E), and 50.0 U/mL Penicillin/ Streptomycin (Gibco Cat#: 15070–063). The medium was stored at 4 °C for up to 2 weeks. The 10%FBS-5bFGF medium consisted of DMEM with 1.0 g/L glucose, 10% FBS, 5.0 ng/mL bFGF, 2.0 mM L-Glutamine and 50.0 U/mL Penicillin/ Streptomycin. The medium, excluding the bFGF, was stored for up to two weeks at 4 °C. To analyze the culture media, the bFGF concentration was analyzed using a bFGF ELISA Kit (Sigma Cat# RAB0182). The glucose and lactate concentrations were analyzed using Yellow Springs Instrument 2900D Biochemistry Analyzer.

### Static culture of eCB-MSCs

For the static culture, the eCB-MSCs were expanded in 75cm^2^ T-flasks (Falcon Cat#: 353136) at an inoculation density of 5000 cells/cm^2^, with 12 mL of media, in a humidified incubator (37 °C and 5% CO_2_ in ambient air). Once the cells neared confluence (~ 80%), they were harvested by exposing the cells to 0.25% Trypsin for 5 min in a humidified incubator (37 °C and 5% CO_2_ in ambient air), followed by deactivation of the Trypsin using FBS containing media. The cells were then enumerated on a haemocytometer using 0.1% Trypan Blue exclusion, and either passaged onto new T-flasks, inoculated on microcarrier beads within bioreactors, or cryopreserved in Cryostor CS10 freezing media (BioLife Solutions Cat # 210102) for future cell characterization.

### Microcarrier preparation

Prior to inoculation, microcarriers (see below for types used) were hydrated in 50.0 mL of 1X PBS (without calcium or magnesium), with 50 U/mL penicillin/streptomycin for 24 h in Erlenmeyer flasks pre-coated with Sigmacote (Sigma, Cat # SL2), to prevent the microcarriers from adhering to the flask surface. The microcarriers were then rinsed with PBS and sterilized by autoclaving before inoculation into the bioreactors with culture media.

### Microcarrier screening in 6 well plates

Initial microcarrier screening was performed in 6-well plates to investigate eCB-MSC attachment to five different microcarriers: Cytodex 1 (GE Healthcare Cat# 17–0448-01), Cytodex 3 (GE Healthcare Cat# 17–0485-01), Cultispher S (Sigma Cat# M9043), Enhanced Attachment (Corning Cat# 3779) and Synthemax II (Corning Cat# 3781). The cells and microcarriers were inoculated into the wells at 6700 cells/cm^2^ (microcarrier surface area) with 3.0 mL of 30%FBS-0bFGF medium. The 6-well plates were placed on a shaking platform (Scientific Excella e5) at 60 rpm with a ¾” diameter shaking orbit and cell attachment counts were performed at 1, 2, 3, 4 and 24 h.

### Bioreactor culture of eCB-MSCs

Two different scales of bioreactors were used in this study - 10 mL microbioreactors (HexaScreen, Barcelona, Spain) and 125 mL spinner flask bioreactors (NDS Technologies, NJ, USA). The 10 mL bioreactors were only used for microcarrier screening. All bioreactors were coated with Sigmacote and autoclaved prior to use. The 125 mL bioreactors were inoculated with 2 g/L microcarriers and culture media at 50% of the final working volume. After 24 h, cells were inoculated into the bioreactors in culture media at 60% of the final working volume. The remaining culture media was added on Day 1 to achieve 100% of the working volume (100 mL). Adequate mixing did not occur at lower volumes than 80% of the working volume in the 10 mL bioreactors. Therefore, the media and microcarriers were added to the bioreactors at 80% of the working volume, and after 24 h the cells were added in media in 100% of the working volume. Unless otherwise specified, the 125 mL bioreactors were inoculated at 5000 cells/cm^2^, and the 10 mL bioreactors were inoculated at 6700 cells/cm^2^. All bioreactors were placed on a magnetic stir plate in a humified incubator (37 °C and 5% CO_2_). Unless otherwise stated, the 125 mL bioreactors were run at 40 rpm and the 10 mL bioreactors were run at 60 rpm. Samples were removed from the bioreactors for enumeration. An attached cell density was determined by adding 0.1% crystal violet with 0.1 M citric acid to lyse the cells and dye the nuclei, which were then counted.

### Harvesting of eCB-MSCs from microcarriers

#### Harvesting samples

Five different enzymes were tested for detachment potential, Trypsin 0.25% (Gibco Cat. #25200) and Trypsin 0.05% (Gibco Cat. #25300), TrypZean (Sigma Cat. #T3449), TrypLE (Gibco Cat. #12605), and Accutase (Invitrogen Cat. #00–4555-56). Samples were taken from the bioreactors and harvested in conical tubes. For the enzyme screening experiments, an exposure time of 9 min was used, then for the following experiment analyzing exposure times, time points of 3, 6, 9, 12, and 15 min were used. The cell suspension was then filtered through a 70 μm sieve (Falcon Cat.# 352,350) and cells were enumerated on a haemocytometer using 0.1% trypan blue exclusion. The harvesting efficiencies were calculated by dividing the number of cells recovered to the attached cell density number that was obtained using the crystal violet nuclei method.

#### Harvesting bioreactors

For the harvest of a 125 mL bioreactor, agitation was suspended and the microcarrier were allowed to settle. Culture media was removed and enzyme was added to the bioreactor, and incubated at 37 °C and 5% CO_2_ for 9 min at an agitation rate of 50 rpm. The cell suspension was then filtered through a 70 μm sieve and enumerated on a haemocytometer using 0.1% trypan blue exclusion. Harvesting efficiency was calculated as previously described.

### Developed process for the expansion of three cell donors

The developed process was used to expand cells from two new Donors (1201 and 1412) and compared to the original Donor (1409). eCB-MSCs were inoculated into separate 125 mL bioreactors, using 2 g/L Cytodex 3 at 5000 cells/cm^2^. They were expanded for 6 days at 40 rpm in 37 °C using the new medium, with the addition of 5 ng/mL bFGF on Day 2. Full bioreactors were harvested on Day 6 using Trypsin 0.25% for 9 min, with continuous agitation at 50 rpm, followed by filtration using a 70 μm sieve. The eCB-MSCs were then passaged into new 125 mL bioreactors using the same expansion conditions as the first passage and harvested after 7 days, then frozen for future cell characterization analysis.

### Cell characterization: Flow cytometry

Donors 1201 and 1409 were further analyzed by flow cytometry to evaluate surface marker expression from static and bioreactor culture conditions. Cryopreserved eCB-MSCs from both conditions were thawed and counted, and subsequently resuspended in flow buffer (1X PBS, 5 mM EDTA (ThermoFisher Cat. 15,575,020), 1% horse serum (Sigma Cat. H0146), and 0.1% sodium azide (Fisher Scientific Cat. S227I-100)). A minimum of 100,000 cells were evaluated per antibody. The following antibodies were used to evaluate the cells: APC anti-human CD29 (BioLegend, Clone: TS2116, Cat. 303,007), Mouse anti-horse CD44:FITC (Biorad, Clone: CVS18, Cat. MCA1082F), Mouse anti-rat CD90 (BD Pharmingen, Clone: OX-7, Cat. 554,895), Mouse anti-human CD105:FITC (Pharmingen, Clone: 266, Cat. 561,443), Mouse anti-horse CD4:FITC (Biorad, Clone: CVS4, Cat. MCA1078F), Mouse anti-horse CD8 (Biorad, Clone: CVS8, Cat. MCA2385F), Mouse anti-horse CD11a/18:FITC (Biorad, Clone: CVS9, Cat. MCA1081F), PE Mouse anti-human CD73 (BD Pharmingen, Clone: AD2, Cat. 561,258), Mouse anti-human CD45 (WSU Monoclonal Antibody Center, Clone: DH16A, Cat. 113,097), Mouse anti-horse MHC I:FITC (Biorad, Clone:CVS22, Cat. MCA1088F), and Mouse anti-horse MHC II:FITC (Biorad, Clone:CVS20, Cat. MCA1085F). Goat anti-mouse IgG1-FITC (Abcam, Cat. 97,239) was used as a secondary antibody against unconjugated primary antibodies. 7-AAD (Sigma Cat. SML1633) was used as a dead cell stain. Negative control samples were cells incubated with no antibody (unstained) and cells incubated with isotype-matched nonbinding primary antibody plus fluorescent secondary antibody. Samples were run and analyzed on a BD Accuri™ C6 (BD Biosciences, San Jose, CA).

### Cell characterization: Tri-lineage differentiation

As with flow cytometry analysis, eCB-MSC cultures from static and bioreactor conditions were thawed and expanded in T-flasks until the appropriate cell number was reached. Osteogenesis and adipogenesis: 6-well plates were seeded at 5000 cells/cm^2^ in MSC expansion medium containing bFGF and grown to 80% confluency. Commercial osteogenic differentiation medium (BulletKit; Lonza, Walkersville, MD, USA Cat. PT-3002) was used to induce osteogenesis in half of the wells (the remaining wells were cultured in MSC expansion medium containing bFGF as a negative control). Adipogenic induction medium consisted of DMEM-LG (Lonza), 15% rabbit serum (Cat. R9133), 500 μM 3-isobutyl-1-methylxanthine (Cat. I5879), 2 mM L-glutamine, 1x antibiotic/antimycotic solution (Cat. A5955), 1 μM dexamethasone (Cat. D2915), 10 μg/ml human insulin (Cat. I9278), 200 μM indomethacin (Cat. I7378) (all from Sigma). Media was changed three times per week and differentiation was terminated after 14 days. To verify osteogenic and adipogenic induction, wells were stained with Alizarin Red and Oil Red O, respectively, as previously described [2].

Chondrogenesis: Chondrogenic induction was performed in pellet culture as previously described [27]. Media was changed three times per week and induction was terminated after 21 days. Pellets were fixed in 10% formalin overnight, then sectioned at 5 μM and stained with toluidine blue to assess chondrogenic differentiation.

### Statistical analysis

Statistical analysis was performed using GraphPad Prism (v6.0). A 2-way ANOVA was done followed by Tukey’s multiple comparison test for all analyses, except for the flow cytometry analysis, where unpaired t-tests were done. All experiments were run using either two stirred suspension bioreactors (*n* = 2), or two T-flasks (n = 2). Two samples were removed from each vessel, and each sample was analyzed in duplicate. Significance was assigned as *p* < 0.05. All graphs are presented with error bars representing the standard error of the mean.

## Results

### Microcarrier screening

**Fig. 2:**
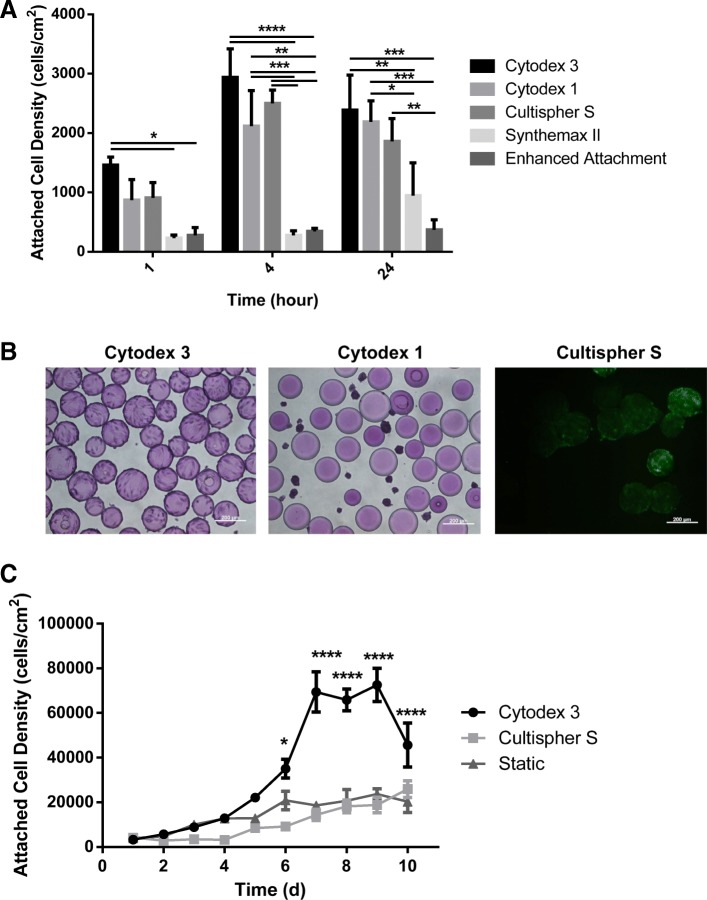
Microcarrier Screening. **a** Attachment over 24 h of eCB-MSCs on Cytodex 3, Cytodex 1, Cultispher S, Synthemax II and Enhanced Attachment microcarriers in 6-well plates. Statistical significance compares the attached cells density at each time point (* *p* ≤ 0.05, ** *p* ≤ 0.01, *** *p* ≤ 0.001, **** *p* ≤ 0.0001) **b** The eCB-MSCs on microcarriers at 24 h. Cultispher S was stained with Calcein-AM/ ethidium homodimer-1, the other microcarriers were stained with crystal violet. Scale bars are 200 μm. **c** Attached cell density over 7 days for Cytodex 3, Cultispher S, and static T-flasks. Error bars are standard error of the mean. Statistical significance compares the attached cells density at each time point

Figure 2a shows the attached cell densities on the five different microcarriers at the different time points screened in 6-well plates. Attachment efficiencies at 24 h were found to be 36% for Cytodex 3, 32% for Cytodex 1, 28% for Cultispher S, 15% for Synthemax II, and 6% for Enhanced Attachment. Cytodex 3 (*p* ≤ 0.001), Cytodex 1 (p ≤ 0.001), and Cultispher S (*p* ≤ 0.01) all had higher attachment efficiencies than Enhanced Attachment at 24 h, while both Cytodex 1 (*p* ≤ 0.05) and Cytodex 3 (p ≤ 0.01) were also higher than Synthemax II at 24 h. The three microcarriers with the highest attachment efficiencies, Cytodex 3, Cytodex 1 and Cultispher S, were then used to expand the cells in 10 mL bioreactors run at 60 rpm over an 8 day period. Figure 2b shows images of the eCB-MSCs on Cytodex 1, Cytodex 3 and Cultispher S at Day 5. By Day 4, the cells had fallen off the Cytodex 1 microcarriers, however the cells were successfully expanded and remained attached on both Cytodex 3 and Cultispher S for the remainder of the 8 day culture period. To quantify the cell expansion, the cells were then expanded in 125 mL bioreactors on Cytodex 3 and Cultispher S over a 10-day period and compared to static growth. As seen in Fig. 2c, higher maximum attached cell densities were observed on Cytodex 3 (*P* ≤ 0.0001) than on Cultispher S or static T-flask culture.

### Bioreactor inoculation phase

#### Cell attachment kinetics

**Fig. 3:**
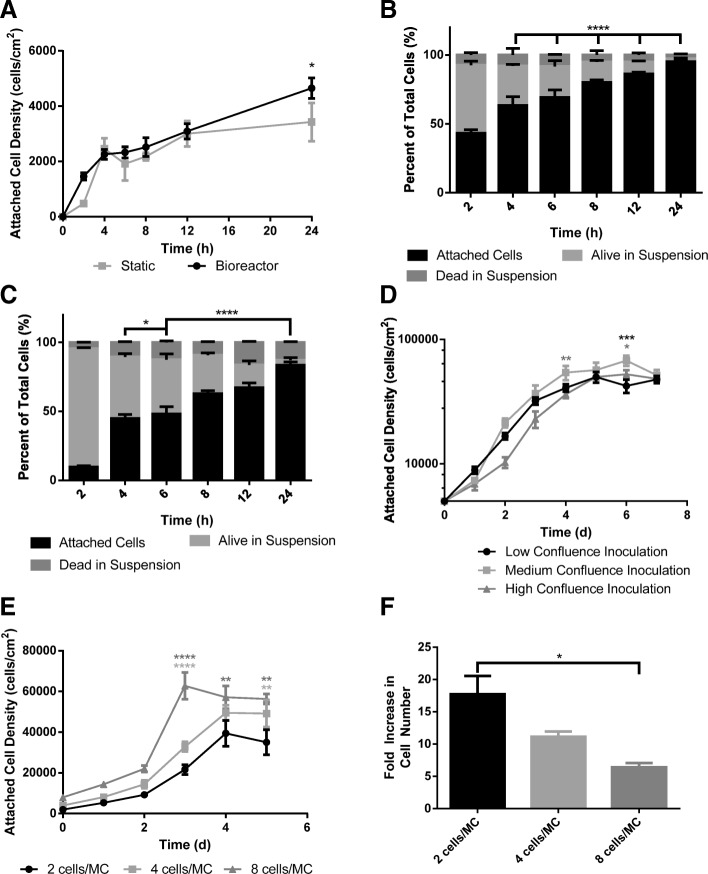
Inoculation Phase. **a** Attachment over 24 h of eCB-MSCs to static T-flasks and Cytodex 3 microcarriers in bioreactors. (* p ≤ 0.05, ** p ≤ 0.01, *** p ≤ 0.001, **** p ≤ 0.0001) **b** Attached cells, alive cells in suspension, dead cells, and total cells of eCB-MSCs over 24 h in bioreactor culture. Statistical significance compares the attached cells to the alive cells in suspension. **c** Attached cells, alive cells in suspension, dead cells, and total cells of eCB-MSCs over 24 h in static culture. Statistical significance compares the attached cells to the alive cells in suspension. **d** Attached cell densities on microcarriers in bioreactors passaged from T-flasks at 3 different densities over a 7 day period. Statistical significance compares the medium inoculation condition to the high and low inoculation conditions. **e** Attached cell densities and **f** Fold increase of eCB-MSCs on microcarriers in bioreactors with inoculation densities of 2 cells/MC, 4 cell/MC and 8 cells/MC. Statistical significance compares the 2 cells/MC condition to the 4 cells/MC and 8 cells/MC conditions

Figure 3a, b and c shows the comparison of cell attachment between eCB-MSCs grown in static T-flasks and on Cytodex 3 microcarriers in bioreactors with a working volume of 125 mL. Up to the 12 h time point, there was similar cell attachment between the static and microcarrier attachment. Between 12 and 24 h, the static attachment plateaued, however the microcarrier attachment continued to increase. At 24 h, there was a higher attached cell density in the bioreactor (*p* ≤ 0.05) than in static culture. In the bioreactor culture, there was a greater number of cells at 24 h than originally inoculated, indicating that cell growth began within the initial 24 h period.

#### T-flask confluency

The eCB-MSCs were expanded in static culture prior to inoculation into bioreactors. Cells from T-flasks with various confluence levels were inoculated into bioreactors at the same inoculation density of 5000 cells/cm^2^ and expanded using 10%FBS-5bFGF medium. The low confluence inoculation bioreactor was inoculated with T-flasks harvested on Day 3, at 20% confluence. The medium confluence inoculation bioreactor was inoculated with T-flasks harvested on Day 4, at 50% confluence. The high confluence inoculation bioreactor was inoculated with T-flasks harvested on Day 5, at 65% confluence. Figure 3d shows the attached cell densities in the bioreactors inoculated with cells from T-flasks at different confluency levels. The bioreactor inoculated from the high confluence T-flask had a longer lag phase than the low and medium confluence T-flasks. On Day 6, the attached cell density in the medium confluence inoculation condition was higher than both the high confluence inoculation condition (*p* ≤ 0.05) and the low confluence inoculation condition (*p* ≤ 0.001).

#### Cell to microcarrier ratio

Three different initial cell-to-microcarrier ratios, 2 cells/MC, 4 cells/MC, and 8 cells/MC, were compared for expansion potential of eCB-MSCs, as seen in Fig. 3e and f. The cells were inoculated into 125 mL bioreactors at various ratios and expanded over 5 days at 40 rpm in 10%FBS-5bFGF medium. The 8 cells/MC inoculation density achieved a higher (*p* ≤ 0.0001) final attached cell density of 63,000 cells/cm^2^, however had the lowest fold increase in cell number of 6.4. The 2 cells/MC inoculation density achieved the lowest attached cell density of 39,000 cells/cm^2^, however had the greatest fold increase in cell number of 17.7.

### Expansion phase

#### Medium development and analysis

**Fig. 4:**
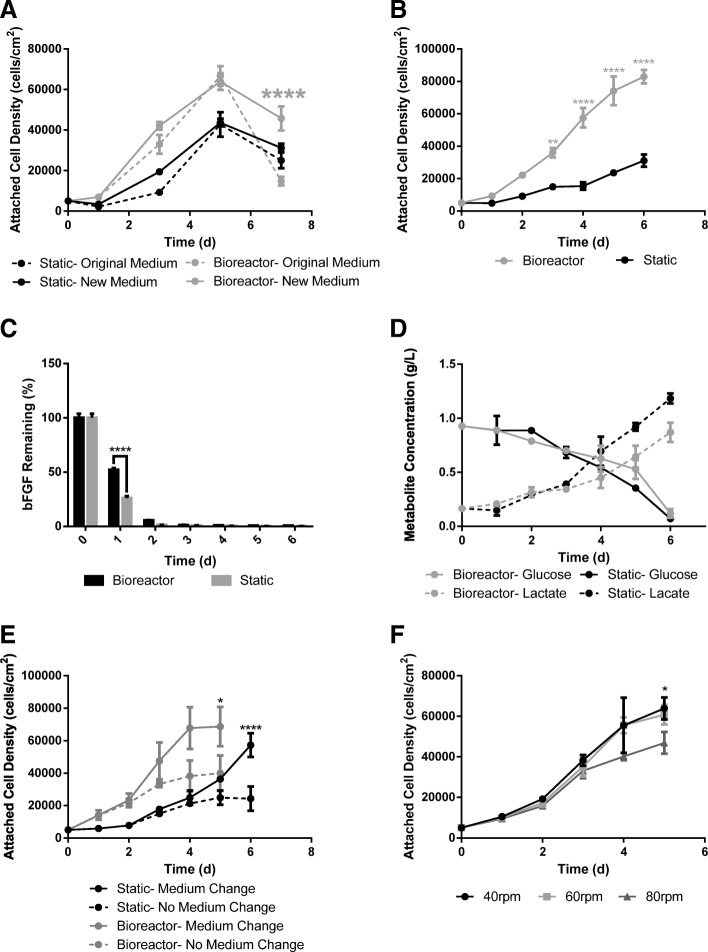
Expansion Phase. **a** Attached cell density over a 7 day period of eCB-MSCs grown in static and bioreactors in new and original medium. Statistical significance compares the new medium to the original medium within the bioreactor and static conditions. (* p ≤ 0.05, ** p ≤ 0.01, *** *p* ≤ 0.001, **** *p* ≤ 0.0001) **b** Attached cell density in static and bioreactor with no medium change over a 6 day period. The medium from the culture was used for media analysis. **c** The percentage of bFGF remaining in the culture medium on each day of expansion in bioreactor and static culture. **d** The concentration (g/L) of glucose and lactate in the culture medium on each day of expansion in bioreactor and static culture. **e** Attached cell density in bioreactor and static culture, with and without medium change. Statistical significance compares the medium change to the no medium change condition within bioreactor and static culture. **f** Attached cell densities of eCB-MSCs grown in bioreactors run at 40 rpm, 60 rpm, and 80 rpm. Statistical significance compares to the 80 rpm condition

The 30%FBS-0bFGF medium (original) was compared to the 10%FBS-5bFGF medium (new) for the expansion of eCB-MSCs in both static and bioreactor culture as seen in Fig. 1a. The only difference between new and original medium occurred on Day 7, in which the new medium achieved higher attached cell densities (p ≤ 0.0001) than the original medium in bioreactor culture.. Based on these results, it was determined that the 10%FBS-5bFGF medium could be used for subsequent studies for the expansion of eCB-MSCs. To determine an appropriate medium replacement regime for the 10%FBS-5bFGF medium, an analysis was done to investigate the glucose, lactate and bFGF concentrations in the medium over the course of a batch culture. Initially the cells were grown in static T-flasks and 125 mL bioreactors as a batch process and media samples and cell counts were performed daily for a 6 day culture period. The attached cell densities can be seen in Fig. 4b. As seen in Fig. 4c, the bFGF concentration in the medium had been nearly depleted by Day 2 in both static and bioreactor culture. The glucose and lactate concentrations can be seen in Fig. 4d. The rate of glucose consumption was calculated to be 2.35 pmol/cell/d in the bioreactor and 7.89 pmol/cell/d in static. The rate of lactate production was calculated to be 3.32 pmol/cell/d in the bioreactor compared to 22.5 pmol/cell/d in static.

Based on these results, the proposed medium replacement regime was the addition of bFGF every 2 days, as well as 50% basal medium change on Day 4 of the culture period. The cells were then expanded in static T-flasks and 125 mL bioreactors, with and without the proposed medium change as seen in Fig. 4e. The effect of the medium change differed between static and bioreactor growth. In the bioreactor culture, when the bFGF was added on Day 2, the expansion of cells greatly increased, and when the 50% medium change was performed on Day 4, the growth plateaued. In static culture, when the bFGF was added on Day 2, there was only a small effect on the cell expansion, and when the 50% medium change was performed on Day 4, the cell expansion greatly increased. There were higher maximum attached cell densities with the medium change condition for both bioreactor culture (*p* ≤ 0.05) and static culture (*p* ≤ 0.0001).

#### Agitation rate

Three different agitation rates - 40, 60 and 80 rpm - were investigated for the expansion of eCB-MSCs in the 125 mL bioreactors. Similar attached cell densities were achieved in both the 40 rpm and 60 rpm bioreactors, with the 80 rpm bioreactor achieving significantly lower attached cell densities (p ≤ 0.05) than the 40 rpm bioreactor by Day 5 as seen in Fig. 4f.

### Harvesting

#### Enzyme type and exposure time

**Fig. 5:**
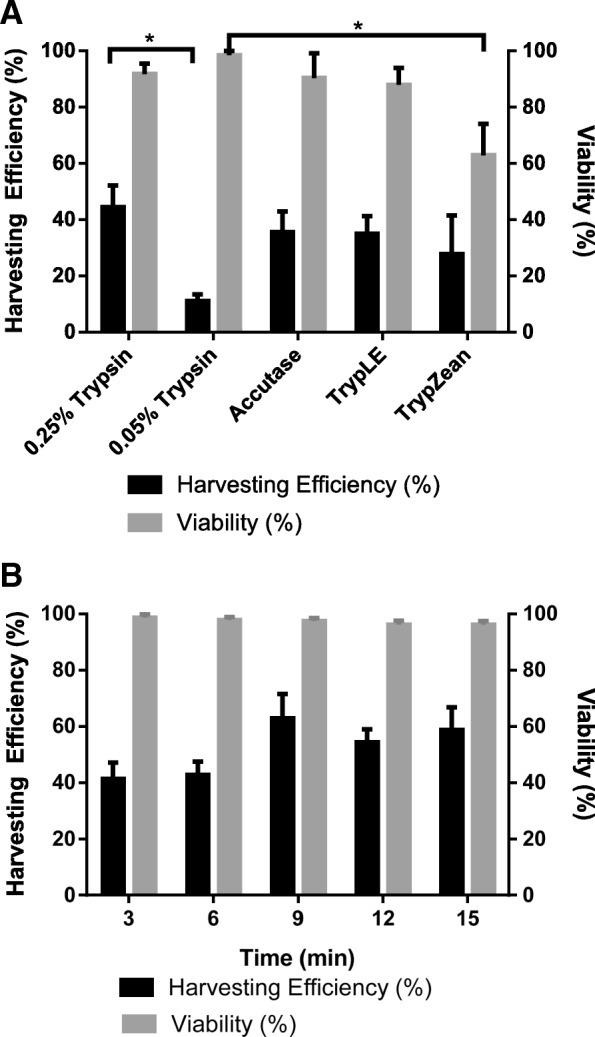
Harvesting Phase. **a** Harvesting efficiencies and viabilities of eCB-MSCs on microcarriers using 0.25% Trypsin, 0.05% Trypsin, Accutase, TrypLE, TrypZean. (* p ≤ 0.05, ** p ≤ 0.01, *** p ≤ 0.001, **** p ≤ 0.0001) **b** Effect of exposure time on harvesting efficiency and viability when harvesting eCB-MSCs on microcarriers using Trypsin 0.25%

Five different enzymes were compared for their removal efficiency of the eCB-MSCs from microcarriers, as seen in Fig. 5a. Viabilities remained above 88% for all enzymes except TrypZean, and all enzymes except 0.05% Trypsin achieved similar detachment efficiencies. The effect of different exposure times on the detachment of eCB-MSCs from Cytodex 3 was tested using 0.25% Trypsin. All viabilities were over 95%, as seen in Fig. 5b, and harvesting efficiencies increased from 3 min to 9 min, and then plateaued, however there were no significant differences.

### Expansion of multiple donors using developed bioprocess

#### Expansion and harvest

**Fig. 6:**
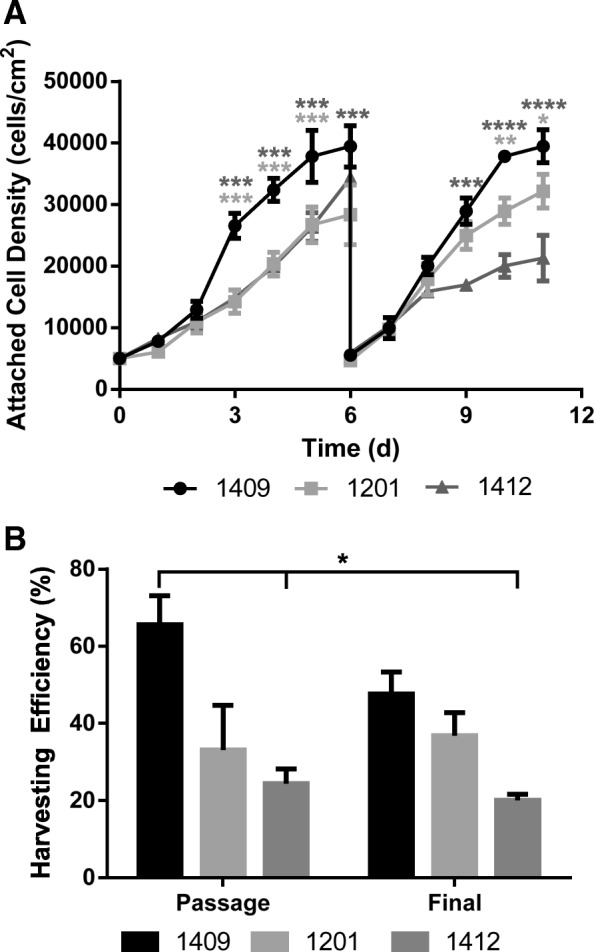
**a** Attached cell densities over two passages for the three different donor cells, 1409, 1201 and 1412 grown in bioreactors. **b** Harvesting efficiencies for the three different donor cells between passages and after the final passage for expansion in bioreactors using the improved harvesting procedure. Error bars represent standard error of the mean. Statistical significance compares Donor 1201 and 1412 each to Donor 1409

The attached cell densities over the two passages are shown in Fig. 6a, and the harvesting efficiencies between passages and at the end of the culture period are shown in Fig. 6b for the three different eCB-MSC lines expanded in stirred suspension bioreactors. The maximum attached cell densities were 40,000 cells/cm^2^, 28,000 cells/cm^2^, and 35,000 cells/cm^2^ for Donor 1409, 1201, and 1412, respectively during the first passage, and 31,000 cells/cm^2^, 32,000 cells/cm^2^, and 21,000 cells/cm^2^ for the second passage. Donor 1409 had higher maximum attached cell densities than Donor 1201 (*p* ≤ 0.001) during the first passage, and had higher maximum attached cell densities than Donor 1201 (*p* ≤ 0.05) and 1412 (*p* ≤ 0.0001) during the second passage. The harvesting efficiencies were 70%, 31%, and 25% for Donor 1409, 1201, and 1412 respectively between passages, followed by 47%, 37%, and 19% for the final harvest. Donor 1409 had higher harvesting efficiencies than Donor 1412 (p ≤ 0.05) for both harvests. The viabilities for all donors, at the end of both passages, were 94% or greater.

#### Surface marker expression and trilineage differentiation

**Fig. 7:**
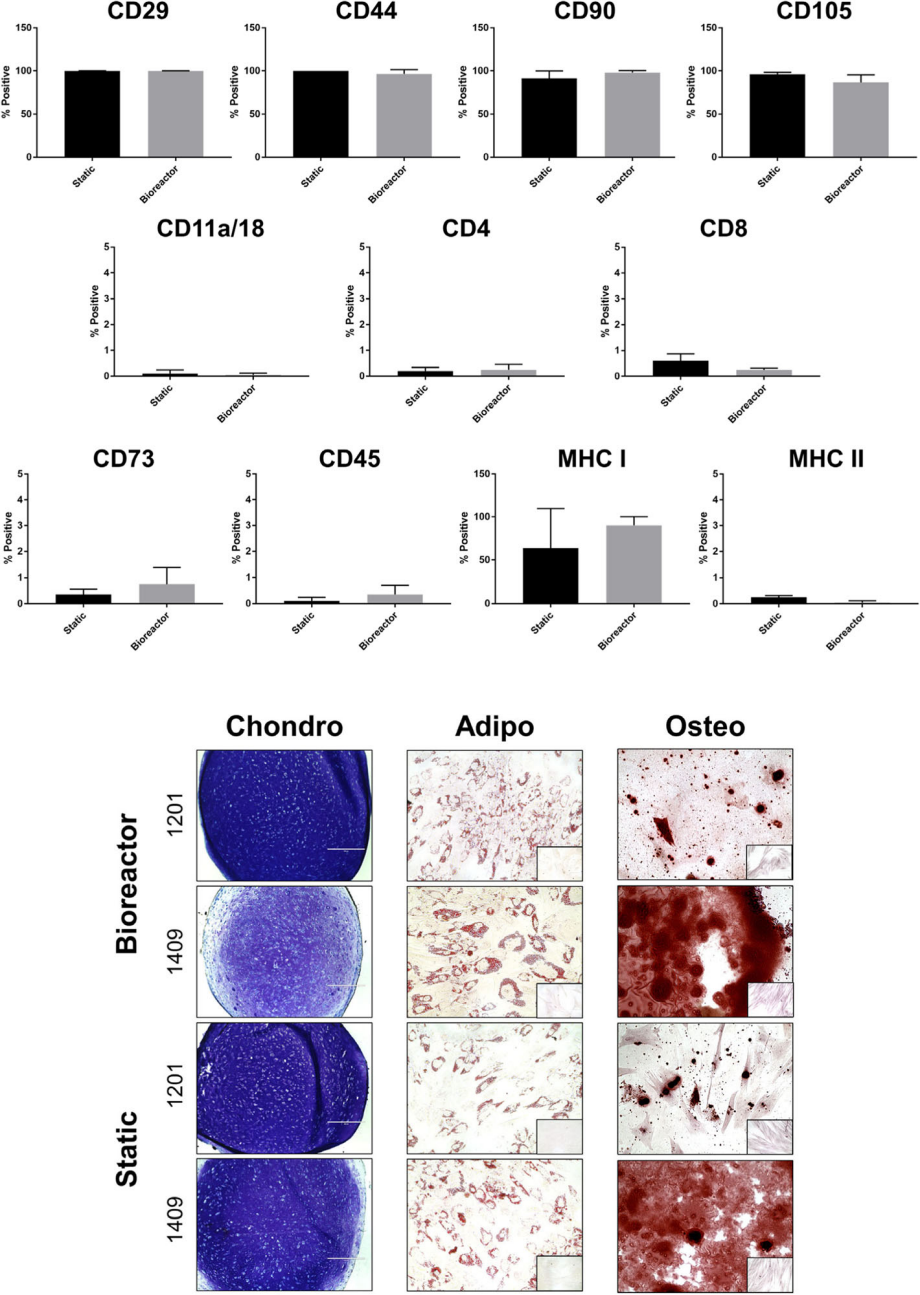
Phenotypic characterization and trilineage differentiation of static and bioreactor-grown eCB-MSCs (donors 1409 and 1201). Top panel: Flow cytometry analysis of MSC and hematopoietic markers shown as a % positive compared to isotype negative control. There were no significant differences between the static and bioreactor for any markers. Bottom panel: Chondrogenic, osteogenic, and adipogenic differentiation of eCB-MSCs. Chondrogenic pellets were sectioned and stained with toluidine blue after 21 days of differentiation. Osteogenic and adipogenic induction was performed for 14 days, then cells were stained with Alizarin Red and Oil Red O, respectively. Insets: Negative controls of osteogenic and adipogenic induction containing only MSC expansion medium and stained with Alizarin Red and Oil Red O, respectively

Donors 1409 and 1201 were subsequently analyzed by flow cytometry and trilineage differentiation to determine if there were any differences in phenotype and/or function as a result of expansion in bioreactor culture. MSCs grown in static and bioreactor culture expressed similar levels of equine MSC markers CD105, CD29, CD44, CD90, and MHC I, with low or absent expression of hematopoietic markers CD4, CD8, CD11a/18, CD45, CD73, and MHC II (Fig. 6). There were no differences between static and bioreactor culture for any of the markers. Osteogenic, adipogenic, and chondrogenic induction of static and bioreactor cultures revealed no differences in differentiation capacity between the two culture systems, though we observed differences in staining intensity between the two donors for chondrogenesis and osteogenesis (Fig. 7).

## Discussion

This pilot study was the first known study to expand equine cord blood MSCs on microcarriers in stirred suspension bioreactors. The first step in the process development was to find an appropriate microcarrier to facilitate attachment as well as growth of the eCB-MSCs. Five microcarriers that are commonly used for the expansion of human MSCs were tested. Both Synthemax II and Enhanced attachment microcarriers had very low attachment of eCB-MSCs. These are both polystyrene microcarriers with proprietary coatings. Cytodex 1, an uncoated microcarrier with a dextran matrix, allowed for cell attachment but not long term expansion, which may have been due to the lack of coating which prevented expansion and spreading of the cells on the microcarrier surface. Cell attachment as well as expansion was facilitated on Cytodex 3, a gelatin coated microcarrier with a dextran matrix, as well Cultispher S, a gelatinous macroporous microcarrier. However, cells on Cytodex 3 achieved higher attached cell densities, likely due to poor nutrient and oxygen transfer into the pores of the Cultispher S microcarrier causing increased cell death. These results were not unexpected as Cytodex 3 is commonly used to expand various sources of MSCs, obtaining high cells densities over long term culture period [10, 11, 17–19, 28–31]. Based on these results, Cytodex 3 was chosen for use in the remaining process development.

Several different inoculation process parameters were investigated for the attachment and expansion of eCB-MSCs on microcarriers in bioreactors. Initially the cell attachment kinetics were investigated and attachment of cells to microcarriers was compared to attachment to static T-flasks. Compared to static attachment, cell attachment to microcarriers occurred much more rapidly, with nearly 50% attachment occurring within the first 2 h, compared to less than 10% in static attachment. The attachment may have been enhanced due to shear effects on the cells promoting cell attachment, as low levels of shear have been found to affect proliferation as well as cytokine production of MSCs [32]. The static flasks were also not coated, while the microcarriers were coated in gelatin, which could have led to the enhanced attachment in bioreactor culture. Additionally, it was observed that the cells in static culture undergo a lag phase, while in the bioreactor a lag phase was absent.

Other inoculation phase conditions were also found to affect cell growth. One such condition was the confluency of the T-flask prior to inoculation into the bioreactors. It was found that with T-flasks at low confluency, a lag phase was not observed, however a lag phase was observed when a T-flask at high confluency was used for inoculation. The cells in the low confluency T-flask were in the exponential phase of growth, while the cells in the high confluency T-flask were in the stationary phase of growth, likely contributing to the lag phase observed using this T-flask confluency. This was consistent with the findings by Balint et al. (2015), who found that when cells were passaged from T-flask to T-flask at 10–50% confluency they had significantly lower population doubling times and a higher proliferation rate than when cells were passaged at 40–70% confluency [14]. To our knowledge, no study has been performed to analyze the effect of T-flask confluency on subsequent growth in bioreactors.

The last condition investigated in the inoculation phase was the initial cell to microcarrier ratio. Three different ratios were investigated, 2 cells/MC, 4 cells/MC, and 8 cells/MC. The 2 cells/MC ratio had the highest fold-increase in cell number, and the 8 cells/MC ratio had the highest attached cell densities. The actual choice of cell to microcarrier ratio in a bioprocess is dependent on other process constraints. For example, if the cells are very scarce, then the 2 cells/MC density would be chosen as high cell densities are still achieved despite the low inoculation density. However, if the process is time sensitive, or the cost of the medium is a limiting factor, then an 8 cells/MC density would be chosen as the greatest cell densities are achieved, with the same quantity of medium, and is reached one day earlier than using the 2 cells/MC or 4 cells/MC densities.

The 30%FBS-0bFGF medium was compared to the 10%FBS-5bFGF medium and the cell growth kinetics of the eCB-MSCs were similar in both media, therefore the addition of bFGF was an appropriate substitute to lower the amount of FBS in the medium. This is consistent with several studies that have shown that bFGF in culture medium enhances expansion of human MSCs, as bFGF is a cytokine that enhances motility and proliferation of several cell types [33–35]. A study by Ibrahim et al., tested different types of basal media, with 10% FBS, with the addition of either 4 or 10 ng/mL bFGF and found greater expansion with 10 ng/mL bFGF, and found that the bFGF was required for growth [20].

Using the 10% FBS-bFGF medium, a medium replacement regime was developed by analyzing the metabolic activity of the cells in static and bioreactor culture. There were significant differences between the metabolism in the cells in static culture compared to bioreactor culture, with the bioreactor cells having very low metabolic activity. Studies analyzing the metabolism of human MSCs grown in stirred suspension bioreactors have found that the glucose uptake rate varied between 5 and 15 pmol/cell/d [10, 36], comparing to our results of 2.35 pmol/cell/d for the bioreactor culture and 7.89 pol/cell/d for the static culture. Studies have also found that the lactate consumption rate varied between 12 and 25 pmol/cell/d [10, 36], compared to our results of 3. 32 pmol/cell/d for the bioreactor culture and 22.5 pmol/cell/d for static culture. However, no studies could be found for the metabolic activity of equine MSCs, and it has been found that human MSCs have different metabolic activity than certain species of animal MSCs [37].

The difference in metabolic activity between static and bioreactor culture could be due to the mechanism by which the MSCs convert glucose to energy. There are two main mechanisms in which MSCs convert glucose to energy (ATP): glycolysis and oxidative phosphorylation. In oxidative phosphorylation, glucose is metabolised to generate ATP with the consumption of oxygen. This is a very efficient method of energy production, with 1 mol glucose generating ~ 36 mol ATP. In glycolysis, glucose is converted to ATP inefficiently, with 1 mol glucose generating ~ 2–4 mol ATP [37–39]. The yield of lactate to glucose was 2 .9g/g in static culture, and 1. 42 g/g in bioreactor culture. Glycolysis may have been occurring in the cells grown in static culture causing the increased glucose consumption, while oxidative phosphorylation may have been occurring in the cells grown in the bioreactor, allowing for a lower glucose consumption while still generating a large amount of energy [37–40]. An increased oxygen concentration due to the agitation occurring in the bioreactors could have caused the cells in the bioreactor to undergo oxidative phosphorylation rather than glycolysis. The diffusion of nutrients through the bioreactor due to the mixing could also alter the metabolic activity of the cells.

Based on the analysis of glucose and lactate in the medium, a medium replacement regime of a basal medium change of 50% on Day 4, and an addition of bFGF every two days was proposed. Common replacement regimes used in a bioreactor process include, 25% daily or every 2 days, 50% either daily, every 2 days or every 3 days, full medium change every 2 days or 3 days or a perfusion (continuous replacement) regime. However, typically no specific analysis is performed to quantify which specific nutrients are limiting, or if any toxic by-products have built up.

The proposed medium change was used to expand the eCB-MSCs in static and bioreactor culture. Differences were again observed between the cells expanded in static and bioreactor culture. The cells in the bioreactor culture were greatly influenced by the bFGF addition, while the cells in the static culture were greatly influenced by the 50% medium change. This could be related back to the glucose consumption rate, which was observed to be much higher in the static expanded cells, therefore required a higher glucose concentration in the media. This demonstrates differences between bioreactor and static expanded cells, and the need for a custom medium replacement regime for the different modes of expansion.

When cells are expanded using microcarrier-based processes, the agitation must be high enough to maintain cells in suspension. However, studies have also shown that higher agitation rates can achieve greater cell expansion, due to improved nutrient and oxygen transfer, as well as shear stresses can trigger cellular responses through mechanotransduction that can enhance proliferation of cells [41, 42]. Three different agitation rates, 40 rpm, 60 rpm and 80 rpm, were compared for cell proliferation in the 125 mL bioreactor. The average shear stress in 125 mL bioreactors have previously been calculated in our lab to be 0.004 Pa, 0.006 Pa, and 0.008 Pa for bioreactors run at 40 rpm, 60 rpm, and 80 rpm. These values are considerably lower than shear stresses that have been found to damage cells (1.5–3 Pa [43]), or to alter cell behavior (0.1–1 Pa [44, 45]). However, the maximum shear stresses, occurring at the tip of the impeller, have been found to nearly 40 times greater than the average shear stress, which is within range to alter cell behavior, and could have contributed to the lower final attached cell densities in the 80 rpm bioreactor.

The harvesting stage of a microcarrier process is very important to detach the cells from the microcarriers and filter to achieve a pure, highly viable cell suspension. Enzymatic removal is the most common method of removing cells from microcarriers, however, the type of enzyme to use is process and cell specific. Therefore, this study investigated five different types of enzymes for detachment efficiency and found similar detachment efficiencies using 0.25% Trypsin, Accutase, TrypLE, TrypZean, and lower efficiency with 0.05% Trypsin, which has a much lower activity level than the other four enzymes. Goh et al. [11] (2013) compared the kinetics of cell detachment using 0.25% Trypsin, TrypLE Express and Type I collagenase and showed that 0.25% Trypsin resulted in the highest cell detachment, as well as higher osteogenic potential compared to TrypLE Express and Type I collagenase. A similar study by Weber et al. (2007) investigated harvesting of human MSCs using 0.25% Trypsin, Accutase, collagenase or a Trypsin-Accutase mixture [46]. Trypsin and Trypsin-Accutase mixtures achieved the highest cell yields and viabilities.

As 0.25% Trypsin was the standard enzyme used to detach eCB-MSCs from static culture, and was successful in removing the cells from the microcarriers, this enzyme was chosen for use in the bioreactor process. The ideal exposure time in the range of 3–15 min was investigated, and it was found that after 9 min the detachment plateaued, therefore this time was chosen for all other experiments. Throughout the harvesting experiments, generally low harvesting efficiencies were observed, despite images showing that the majority of cells had detached. Upon further investigation, it was found that many cells had been trapped in the sieve used for filtering. As the surface area of the sieve was small, compared to the number of microcarriers being filtered, a microcarrier cake built up on top of the sieve, preventing cells from passing through. A sieve with a larger filter area would be advantageous to achieve higher harvesting efficiencies.

Using the developed process, cells from two new donors were compared to the original cell donor for expansion over two passages in the 125 mL bioreactors. The cell densities of Donor 1201 increased slightly between passages, while those from Donor 1409 and Donor 1412 decreased between passages. It is possible that the growth of the eCB-MSCs using our process could have selected for a certain sub-population of cells in Donor 1201, therefore when the cells were passaged, the cells reached greater maximum attached cell densities during the second passage. All the cells were grown at a high passage, specifically donors 1409 and 1412 which were at passage 10 during the first passage in the bioreactor, and passage 11 in the second passage. Some stem cells have been found to reach senescence at high passages. A study by Bonab et al. (2016), found that population doubling times of human BM-MSCs increased substantially during the 10th passage of cells [47]. This could have been attributed to the decrease in cell growth between the two passages. Variability in proliferation potential between donors has previously been observed in both human [48, 49] and equine MSCs [50, 51]. Heathman et al (2016), and Phinney et al. (1999), compared human BM-MSCs donors for proliferation potential in static and found up to a 12-fold difference between donors. Donor to donor variability has also been shown in equine MSCs, with a study by Carter-Arnold et al. (2012) showing high variability in proliferation between 6 different equine BM-MSC donors.

There was variability in not only the expansion of the eCB-MSCs between donors, but also in the harvesting. Donor 1409 cells, which the harvesting protocol was developed for, achieved the highest harvesting efficiencies, followed by Donor 1201 and 1412. It was shown in the kinetic growth data of the cells, that there were differences in the cells from different donors, therefore this could have resulted in differences in efficiency of the enzymatic harvesting procedure. The low harvesting of all donors can be attributed to the filter as discussed earlier.

The donors used in this study were from cells from two different breeds of horses: Quarter horses and Thoroughbreds, as well as both male and female. To decrease donor to donor variability, the process may need to be altered to account for different breeds and/or sexes. However, if an allogenic treatment is utilized, several prospective donors can be screened for proliferation potential, or for other desirable properties such as chondrogenic potential, and only certain donors can be chosen to be used for treatment.

Maximum attached cell densities of 75,000 cells/cm^2^ were achieved when expanding eCB-MSCs in stirred suspension bioreactors. No other published papers were found that expanded eCB-MSCs in stirred suspension bioreactors, while only one study was found for human cord blood MSCs expanded in stirred suspension, in which cell densities of 45,000 cells/cm^2^ were reached [18]. Other studies expanding various sources of MSCs on Cytodex 3 achieved attached cell densities ranging from 40,000–70,000 cells/cm^2^, comparable with our results [28, 29].

The required number of cells to treat a patient (approximately 10^9^ Ref [7]), could be achieved with a 2 .5L bioreactor. However, it is expected that if these cells were grown in computer-controlled bioreactors, controlling the dissolved oxygen and pH, even greater attached cell densities could be achieved, decreasing the required volume. Comparably, to achieve 10^9^ MSCs in static culture, a 40-layer CellSTACK® would be required, which uses twice the medium volume as a 2 .5L bioreactor, greatly increasing the cost. As well, it would not be possible to control the dissolved oxygen and pH in the CellSTACK® system, thus oxygen and nutrient gradients could occur, affecting cell growth as well as producing a less homogenous product.

The surface marker expression and trilineage differentiation capacity of the eCB-MSCs did not differ between static and bioreactor culture, consistent with a previous report comparing these two expansion methods in human MSCs [52]. The surface markers assessed have been extensively used to characterize equine MSCs, as they appear to be mostly constitutively expressed/not expressed among MSCs from various sources and at different passage numbers [53–56]. Reports on CD105 and MHC I expression are variable, however we did not observe a difference in expression between culture systems. While there was variability in chondrogenic pellet Toluidine Blue staining and Alizarin Red staining for osteogenesis, both donors evaluated showed capacity for trilineage differentiation at later passages (passage 11). This is likely as a result of the addition of bFGF to the culture media [34]. More characterization is needed to ensure that the immunomodulatory potency and in vivo function of the eCB-MSCs remains unchanged between culture systems.

## Conclusions

The use of mesenchymal stem cells as a treatment option for musculoskeletal injuries is becoming increasing popular. However, due to the large number of cells required for treatment, a large scale expansion process is required to efficiently obtain a clinically relevant number of cells. Expansion of MSCs in stirred suspension bioreactors using microcarriers as a scaffold has the potential to produce clinically relevant cell numbers while increasing cell quality and decreasing the risk of contamination, labour and monetary requirements. The process for the expansion of eCB-MSC on microcarriers in stirred suspension bioreactors was developed in three different phases, 1) Inoculation Phase, 2) Expansion Phase, 3) Harvesting Phase. Using the developed process, three different donors of eCB-MSCs were successfully expanded while maintaining their phenotype and differentiation capacity, thus demonstrating the ability for eCB-MSCs to be expanded in stirred suspension bioreactors to obtain clinically relevant number of cells.

## Abbreviations

ATP: Adenosine triphosphate
bFGF: Basic fibroblast growth factor
BM-MSC: Bone marrow mesenchymal stem cell
CB-MSC: Cord blood mesenchymal stem cell
DMEM: Dulbecco modified eagles medium
eCB-MSC: Equine cord blood mesenchymal stem cell
ELISA: Enzyme linked immunosorbent assay
FBS: Fetal bovine serum
MC: Microcarrier
MSC: Mesenchymal stem cell
PBS: Phosphate buffered saline
